# Grammatical evolution decision trees for detecting gene-gene interactions

**DOI:** 10.1186/1756-0381-3-8

**Published:** 2010-11-18

**Authors:** Alison A Motsinger-Reif, Sushamna Deodhar, Stacey J Winham, Nicholas E Hardison

**Affiliations:** 1 Bioinformatics Research Center, North Carolina State University, Raleigh, NC, USA 27695; 2 Department of Statistics, North Carolina State University, Raleigh, NC, USA 27695; 3 Department of Computer Science, North Carolina State University, Raleigh, NC, USA 27695

## Abstract

**Background:**

A fundamental goal of human genetics is the discovery of polymorphisms that predict common, complex diseases. It is hypothesized that complex diseases are due to a myriad of factors including environmental exposures and complex genetic risk models, including gene-gene interactions. Such epistatic models present an important analytical challenge, requiring that methods perform not only statistical modeling, but also variable selection to generate testable genetic model hypotheses. This challenge is amplified by recent advances in genotyping technology, as the number of potential predictor variables is rapidly increasing.

**Methods:**

Decision trees are a highly successful, easily interpretable data-mining method that are typically optimized with a hierarchical model building approach, which limits their potential to identify interacting effects. To overcome this limitation, we utilize evolutionary computation, specifically grammatical evolution, to build decision trees to detect and model gene-gene interactions. In the current study, we introduce the Grammatical Evolution Decision Trees (GEDT) method and software and evaluate this approach on simulated data representing gene-gene interaction models of a range of effect sizes. We compare the performance of the method to a traditional decision tree algorithm and a random search approach and demonstrate the improved performance of the method to detect purely epistatic interactions.

**Results:**

The results of our simulations demonstrate that GEDT has high power to detect even very moderate genetic risk models. GEDT has high power to detect interactions with and without main effects.

**Conclusions:**

GEDT, while still in its initial stages of development, is a promising new approach for identifying gene-gene interactions in genetic association studies.

## Background

In the last decade, the field of human genetics has experienced an unprecedented burst in technological advancement, allowing for exciting opportunities to unravel the genetic etiology of common, complex diseases [1]. As genotyping has become more reliable and cost-effective, genome-wide association studies (GWAS) have become more commonplace tools for gene mapping, allowing hundreds of thousands or millions of genetic variants to be tested for association with disease outcomes [1]. Typically, traditional statistical approaches (i.e. *χ*^2 ^tests of association, regression analysis, etc.) are used to test for univariate associations, and then those associations are evaluated for replication and validation in independent patient cohorts [2]. This traditional approach has been very successful in identifying strong single gene effects in many common diseases [3], but limitations of this traditional approach have become a focus as the variation explained by these single loci do not come close to the estimates of variance explained by genetics (heritability) known for many diseases [4].

This unexplained variation is hypothesized to be due to more complex etiologies underlying complex diseases [5]. These complex mechanisms include rare variants with high penetrance, locus heterogeneity, and epistasis. In particular, the ubiquitous nature of epistasis, or gene-gene and gene-environment interactions, in the etiology of human diseases presents a difficult analytical challenge [5]. Traditional statistical approaches are limited in their ability to detect interaction models due to their reliance on hierarchical model building strategies and concerns with high dimensional data (including the curse of dimensionality) [6]. These limitations have been previously described in detail [7]. In response to these limitations, many novel data-mining approaches have been developed [8]. The majority of these methods rely on either a combinatorial search approach (such as Multifactor Dimensionality Reduction [9], Combinatorial Partitioning Method [10]) or on a hierarchical model building strategy (such as with Random Forests™[11]). The combinatorial approaches are ideal for detecting purely interactive effects (with no single-locus main effects), but are too computationally intensive to detect higher order interactions in large datasets (such as GWAS). The hierarchical approaches are often computationally efficient, but are unable to detect purely epistatic effects [8]. Methods are needed that can detect pure epistatic models in the absence of main effects with realistic computation time. Additionally, as the goal of such data-mining analysis is best described as "hypothesis generation" as opposed to traditional "hypothesis testing," such methodologies need to generate understandable, interpretable models that can be evaluated in follow-up studies [12]. Both replication and functional studies are crucial for the translation of such bioinformatics models.

The use of evolutionary computation (EC) algorithms is one potential solution to these concerns, and has previously shown success in genetic association studies [8]. Several EC algorithms (including genetic algorithms (GA), genetic programming (GP), and grammatical evolution (GE)) have been used to optimize a range of classifiers (neural networks, naïve Bayes classifiers, etc.) to detect complex genotype/phenotype associations. While the success of these methods has been promising, there have been limitations in the interpretability of these models. Specifically, GE optimized neural networks (GENN) have been highly successful in a range of real and simulated data [13], but the resulting neural network models are "black box" models that are difficult to interpret, and are often passed to post hoc "white box" modeling for evaluation [13]. To overcome this problem, we propose using grammatical evolution to build "white box" models that are readily, immediately understandable. Similar approaches have been successful in other fields [14-16], strengthening the hypothesis that this approach would be successful in human genetics. Additionally, similar machine learning approaches have been shown to be successful in genetic applications. Methods such as Symbolic Data Analysis (SDA) [17], a computational evolution system (CES) [18], and Ant Colony Optimization (ACO) [19] have been successfully applied to genetic applications. Specifically, we use grammatical evolution to optimize decision trees for analysis of genetic association studies. In the current manuscript, we introduce our Grammatical Evolution Decision Tree (GEDT) approach and software. We then demonstrate the method on a range of simulated gene-gene interaction models, and show that it has high power to detect interactions in a range of effect sizes.

## Results

### Algorithm

#### Grammatical Evolution (GE)

Grammatical Evolution (GE) is a form of evolutionary computing that allows the generation of computer programs using grammars [20]. The modularity of GE makes it flexible and easy to use. GE uses linear genomes and grammars to define populations. In GE, each individual consists of a binary string divided into codons. Mutation takes place on individual bits along this string (or chromosome) and crossover only takes place between codons. An individual phenotype is produced by translating codons according to the grammar. The resulting individual can then be tested for fitness in the population and evolutionary operators can be applied [21].

GE is inspired by the biological process of generating a protein (phenotype) from the genetic material (DNA genotype) through the processes of transcription and translation. By using a grammar to define the phenotype, GE separates genotype from phenotype and allows greater genetic diversity within the population than other evolutionary algorithms [20]. Analogous to the biological process, a variable-length binary string is generated as the "DNA" of the GE process, where a consecutive group of bits is considered to be a single codon. This binary string is then transcribed into an integer string using the binary code with each codon representing an integer value. These integer values are then translated by a mapping function into an appropriate production rule from the grammar definition. An appropriate production rule is selected by the following mapping function:

rule = (codon integer value) MOD (Number of alternatives for the current non-terminal)

These production rules are then applied to a set of non-terminals to generate the terminals of the executable program. In the case that after transcribing the entire genome the production rule is not complete, the genome is wrapped around like a circular list and codons reused. The grammar used for the current application is described below. Details have been previously described [21].

#### Decision Trees

A decision tree is a hierarchical decision-making model that consists of internal decision nodes and terminal leaf nodes[22]. Internal decision nodes represent attributes of an individual whereas leaf nodes represent the class the individual belongs to. The root node either corresponds to an initial criterion or an attribute of an individual. Root and other internal nodes are connected via directed edges so that a hierarchical structure is formed. Each outgoing edge from an internal node corresponds to the value of the attribute that the node represents.

**Figure 1:**
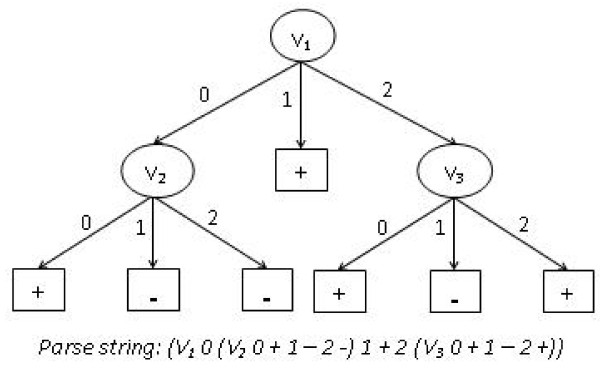
**An example of a decision tree generated by GEDT**. The corresponding parse string for this tree is also shown, which is obtained by using the mapping process. Here, decision nodes V_1_, V_2 _and V_3 _correspond to the SNP attributes of the data. Case and control values are represented as classes '+' and '-', respectively.

Decision trees have been widely used in a variety of applications, such as image classification [23], and pattern recognition [24]. As a learning tool, they offer many advantages that make them ideal for application in human association studies. First, they can model data that has non-linear relationships and/or interactions between variables. Second, they can handle large quantities of data in reasonable computation time. Thirdly, they are very easy to understand and communicate, which is crucial in such a collaborative, interdisciplinary field such as human genetics [12]. From the output model, it is possible to determine what attributes of individuals play an important role in dividing the data in smaller parts and what decisions were made at each internal node. Finally, they are very easy to interpret. Any decision tree can be translated to IF-THEN statements or SWITCH-CASE statements, making it readily human-readable. All these characteristics of decision trees make them a "white-box" model because the way the output is derived from input variables (by going through internal decision nodes) is extremely transparent. This makes them ideal for the "hypothesis generation" motivation of data-mining analysis. An example decision tree is presented in Figure 1.

#### Grammatical Evolution Decision Trees (GEDT)

For the current study, GE has been implemented to optimize decision trees (DTs) to detect gene-gene interactions in genetic association studies. The first step of this implementation was the construction of an appropriate grammar for the mapping of DTs that conform to the problem at hand. For genetic association data, the input variables/attributes represent genotypes at specific loci, where a genotype can take one of three genotype values for a bi-allelic SNP (AA, Aa, aa), encoded as 0, 1, and 2. This encoding makes no genetic model assumptions, so this analysis is both statistically and genetically nonparametric. Additionally, while in the current study we evaluate only genetic input variables, any categorical input variables could also be evaluated to detect gene-environment interactions. The output variable (class variable) can take one of two values, either positive (for cases) or negative (control) states.

The GE process begins with the generation of a large number of randomly generated binary strings that are transcribed into integer strings, and then are translated into DTs using the following grammar.

The grammar can be represented by the tuple {N, T, P, S}, where N is the set of non-terminals, T is the set of terminals, P is a set of production rules that maps the elements of N to T, and S is a start symbol which is a member of N. The following non-terminals were chosen:

N = {S, pseudoV, v, val_0_, val_1_, val_2_, class}

Here, S represents the start codon in the genome. The non-terminal 'pseudoV' is used to represent the tertiary structure of the tree and to allow recursion. Non-terminals 'val_0_', 'val_1_' and 'val_2_' represent the possible values a genetic attribute/variable can have and finally, non-terminal 'class' represents the class an individual belongs to. The following terminals were identified:

T = {0, 1, 2, +, -, V_1-n_}

where the set {V_1_, V_2_, ..., V_n_} represents the variable set which correspond to SNPs in the dataset. Terminals '0', '1' and '2' represent possible values these variables can hold, whereas terminals '+' and '-' represent the class values, which correspond to the case/control values an individual belongs to.

The following production rules were used to define BNF grammar for GEDT:

(1) S := <pseudoV>

(2) pseudoV := <v> <val_0_> <pseudoV> <val_1_> <pseudoV> <val_2_> <pseudoV> | <class>

(3) val_0 _:= 0

(4) val_1 _:= 1

(5) val_2 _:= 2

(6) class := + | -

(7) v := V_1 _| V_n_

where n is equal to the total number of potentially predictive variables/attributes in the dataset. As integer codons are read from the variable-length binary strings, these production rules are used in the mapping function to generate decision trees. The process of generating decision trees can be understood by studying the second production rule of this grammar. The 'pseudoV' non-terminal can be substituted by either a string of seven other non-terminals or 'class', where the latter represents the terminating condition (it also takes care of the cases where all individuals belong to only one class). The first alternative starts with a variable, which is the root of the tree (or sub-tree). It is followed by three values for that variable and each value corresponds to the 'pseudoV' non-terminal. This represents the recursive condition. Now, each of these 'pseudoV' terminals are again substituted in two ways and the process continues until all non-terminals are substituted.

**Figure 2:**
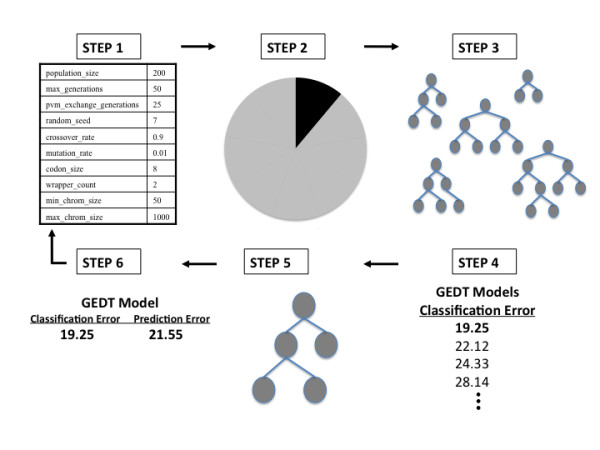
**The GEDT Algorithm**. An overview of the GEDT process that shows the six-step process of initialization, cross-validation, training, fitness evaluation using balanced error, natural selection (tournament) and testing - evaluating prediction error. The steps are as described in the Algorithm section.

After a tree is built using this grammar, the fitness of the DT model is measured, based on how accurately the model classifies all the individuals in the dataset. In order to make our methodology robust to class imbalance (when there is an unequal number of cases and controls in the dataset), we implemented balanced accuracy as the fitness metric [25]. Using this function, poor performance in either class will lead to a poor overall fitness and the evolutionary process will be directed towards a solution that performs well in classifying both the sample classes correctly. The fitness function is calculated as one-half times the addition of ratio of the correctly classified case samples to the total number of case samples and ratio of correctly classified control samples to the total number of control samples. In other words, the fitness measure is equivalent to the arithmetic average of sensitivity and specificity [25]. In the case of balanced data balanced accuracy is the same as classification accuracy. The formula used is shown below:

$$\begin{array}{l} \text{Balanced accuracy}=\left(\text{sensitivity}+\text{specificity}\right)/\text{2}\\ \;\;\;\;\;=½\left\{\left[\text{TP}/\left(\text{TP}+\text{FN}\right)\right]+\left[\text{TN}/\left(\text{TN}+\text{FP}\right)\right]\right\} \end{array}$$

where TP represents true positives, TN represents true negative, FP represents false positives, and FN represents false negatives. While only the balanced case is considered in the current study, this fitness function is robust to class imbalance for future studies or real data analysis. This fitness metric is then used in a genetic algorithm (GA) to automatically evolve the optimal DT for the data at hand. In this GA, individuals with the highest fitness values are more likely to pass on their "genetic material" to the next generation. For GEDT, we use the GE process to evolve every aspect of a decision tree model - including variable selection (which attributes/variables should be included in the model) and the recursive structure of the DT. The steps of GEDT are outlined in Figure 2, and are similar to those previously described for a grammatical evolution optimized neural network strategy [13].

First, GEDT parameters must be initialized in the configuration file, including mutation rate, crossover rate, duplication rate, population size, type of selection, wrapping count, minimum and maximum chromosome size, sensible initialization depth, and number of generations. Second, the data are divided into 10 equal parts for 10-fold cross-validation. 9/10 of the data is used for training, and later the other 1/10 of the data is used to evaluate the predictive ability of the model developed during training [26]. Third, an initial population of random solutions is generated to begin the training process. Sensible initialization is used to guarantee the initial population contains only functioning DTs [20,21]. In the sensible initialization step an expression tree is created using the grammar described above. The software assigns a minimum depth to each rule that describes the depth required for the rule to be completed. As each tree is built, the algorithm randomly selects only rules that can fit within the remaining depth of the tree. Half of the individual DTs are built to the maximum depth by only selecting recursive rules until a non-recursive rule must be chosen to complete the tree and half are generated to a random depth no greater than the maximum by selecting any rule that can fit in the remaining depth of the tree [16,27]. The final step in initialization is to convert nodes of the tree into corresponding codons. Fourth, each individual genome is translated into a DT according to the rules of the grammar described above. Each DT is evaluated on the training set and its fitness (balanced accuracy) is recorded. Fifth, the best solutions (those with the highest balanced accuracy) are selected for crossover and reproduction using user-specified proportions. The selection can be performed in a number of ways such as rank, roulette, tournament, or uniform. We have used tournament selection as it is efficient for parallel architectures and it is easy to adjust its selection pressure to fine-tune its performance [28]. During duplication, some of the best solutions are directly duplicated (i.e. reproduced) into the new generation. During mutation, some other fraction of the best solutions are selected to undergo mutation. Mutation is performed on individual bits and involves flipping of the binary value along the genome. During cross-over, another part is selected for cross-over with other best solutions. It is performed at the chromosomal level. We have used a one-point cross-over operator. After these operators are applied, the new generation is formed, which is equal in size to the original population. The new generation created by a selection technique specified in the configuration file begins the cycle again. This continues until a balanced accuracy of 100% or a user-specified limit on the number of generations is met. An optimal solution is identified after each generation. Periodically, the best solution is replicated between sub-populations. At the end of GEDT evolution, the overall best solution is selected as the optimal DT. Sixth, this best GENN model is tested on the 1/10 of the data left out to estimate the prediction error of the model. Steps two through six are performed ten times using a different 9/10 of the data for training and 1/10 of the data for testing. The goal of GEDT is to find a model that not only fits the data at hand, but will predict on future, unseen data. Cross validation is used in GEDT to prevent overfitting [26]. Overfitting refers to the phenomenon in which a predictive model may well describe the relationship between predictors and outcome in the sample used to develop the model, but may subsequently fail to provide valid predictions in new samples. The use of both classification balanced accuracy and prediction balanced accuracy within the GEDT algorithm emphasizes generalizability of the final model. As described above, for each cross-validation interval, a best model is chosen based on highest accuracy of all models evaluated for that interval - resulting in a total of 10 models (one best model for each interval). A classification accuracy and prediction accuracy are recorded for each of the models and a cross-validation consistency can be measured to determine those variables that have a strong signal in the gene-gene interaction model. Cross-validation consistency summarizes the number of times each variable is present in the GEDT model out of the best models from the ten cross-validation data splits. The higher the cross-validation consistency, the stronger the support for the model. The locus/loci with the highest cross-validation consistency are chosen as the final model of the GEDT analysis.

#### Random Search Decision Trees

As a negative control, a random-search decision tree algorithm was implemented. The random search generates the initial chromosome population as described above for GEDT, but this generation occurs at every generation instead of only at the beginning of the run (such that no cross-over, mutation, or evolution occurs, only a random search. Each genome is converted by the grammar into a decision and the fitness is determined just as it is for GEDT. The algorithm stores the single best tree from all the generations and returns that as the final model. All other networks are discarded.

#### C4.5 Decision Tree Modeling

To compare the performance of GEDT versus a more traditional decision tree approach for the purely epistatic model, the C4.5 algorithm was used to evaluate each of the purely epistatic datasets (described below). The C4.5 algorithm has previously been described in detail [29]. Briefly, the C4.5 algorithm builds decision tree models by selecting attributes (for genetic association studies these are typically SNPs) that most effectively split the test samples into subsets that are enriched for one class or another (for genetic association studies these are case/control classes). The criterion used for evaluating the effectiveness of a split is the normalized information gain (difference in entropy) that results from splitting the data based on the attribute categories. The attribute with the highest gain is selected, and the algorithm recurs on the smaller sub-lists created by this split to hierarchically build a decision tree. Pruning is an important component of traditional decision tree modelling, and is often used in applications of the C4.5 algorithm. In the current study we use subtree replacement pruning [30], where nodes in a tree may be replaced with a leaf (which reduces the number of tests along a certain path). This process starts from the leaves of the fully formed tree, and works backwards toward the root.

### Testing

For the purposes of the current study, purely epistatic genetic models were generated with varying effect sizes. These models emulate the situation where the phenotype under study cannot be predicted from the independent effects of any single gene, but is the result of combined effects of two or more genes [31]. As discussed above, such epistatic (gene-gene interaction) models are increasingly assumed to play an important role in the underlying etiology of common genetic diseases [5]. We used penetrance functions to represent epistatic genetic models, where penetrance defines the probability of disease given a particular genotype combination by modeling the relationship between genetic variations and disease risk. The genetic variations modelled are single-nucleotide polymorphisms (SNPs). For each individual, a total of 100 SNPs were simulated, where two of the SNPs are associated with the outcome, and 98 are noise SNPs. Case-control data was simulated with 125 cases and 125 controls generated for each dataset (representing very small sample sizes), and 100 datasets were generated for each genetic model and effect size combination (described below).

We used a well-described epistasis model exhibiting interaction effects in the absence of main effects, and two interaction models with main effects. Models that lack main effects challenge the method to find interactions in a complex dataset. The models with both marginal and interaction effects may represent more common biological models. The general penetrance functions used for the simulation are shown in Table 1. The first model, based on the nonlinear XOR function, is a modification of the model initially described by Li and Reich [32]. This model generates an interaction effect in which low risk of disease is dependent on inheriting a heterozygous genotype (Aa) from one locus or a heterozygous genotype (Bb) from a second locus, but not both. The second model, called the BOX model, is a symmetric two-locus interaction with main effects at both loci and is a variation on the dominant-dominant model described by Neuman and Rice [33]. In this second model, low risk of disease is dependent on inheriting two low-risk alleles at either one or both loci (AA and/or BB). The third model, referred to as the MOD function, has an asymmetric risk pattern, shown in Table 1. This model represents a modifying effect model on an exclusive OR function described by Li and Reich [32], creating a main effect in addition to the interaction.

**Table 1:** Penetrance patterns for 2-locus epistatic models

Model	XOR			BOX			MOD		
**Genotype**	**AA**	**Aa**	**aa**	**AA**	**Aa**	**aa**	**AA**	**Aa**	**aa**

**BB**	y	x	y	x	x	x	x	y	y

**Bb**	x	z	x	x	y	y	x	x	y

**bb**	y	x	y	x	y	y	y	y	x

Cells marked "x" represent genotype combinations with lower risk. The values "x," "y," and "z" represent penetrance values with 0 < x < y ≤ z < 1 which were chosen to achieve the desired heritability. For XOR models with MAF = 0.5, z = y; for XOR models with MAF = 0.25, z > y to achieve no marginal effects at either locus.

For each of the three genetic models (XOR, BOX, and MOD), five different effect sizes were used, with two different minor allele frequencies (resulting in a total of 30 sets of data simulations). Effect size was measured as the proportion of the trait variance that is due to genetics, or broad sense heritability. As calculated according to Culverhouse *et al *[34], heritabilities for the simulated models ranged from 1-10%, capturing a range of potential models with relatively low effect sizes. Genotypes were generated according to Hardy-Weinberg proportions at two different allele frequencies, 0.25 and 0.5. For the XOR function, these models exhibit interaction effects in the absence of any main effects. For the BOX and the MOD models, both main effects and interactions are seen. A summary of the characteristics of all the models simulated are listed in Table 2. GenomeSim software described by Dudek *et al *[35] was used to simulate the data.

**Table 2:** Power Results for Simulated Models, n = 250

Model Number	Heritability (%)	Minor Allele Frequency	Genetic Model	Power (%)		Power (Lib %)	
				
				GEDT	Random Search	GEDT	Random Search
1	1	0.25	XOR	0	0	1	1

2	1	0.5	XOR	0	0	2	1

3	2.5	0.25	XOR	0	0	1	0

4	2.5	0.5	XOR	0	0	2	0

5	5	0.25	XOR	1	0	2	0

6	5	0.5	XOR	1	1	2	0

7	7.5	0.25	XOR	3	0	4	0

8	7.5	0.5	XOR	2	0	6	0

9	10	0.25	XOR	0	0	1	1

10	10	0.5	XOR	4	0	7	1

11	1	0.25	Box	2	2	28	0

12	1	0.5	Box	5	1	24	0

13	2.5	0.25	Box	13	0	59	0

14	2.5	0.5	Box	16	0	69	1

15	5	0.25	Box	57	0	90	2

16	5	0.5	Box	35	0	82	0

17	7.5	0.25	Box	72	0	95	1

18	7.5	0.5	Box	53	0	93	4

19	10	0.25	Box	83	0	100	2

20	10	0.5	Box	69	1	95	1

21	1	0.25	Mod	1	1	15	3

22	1	0.5	Mod	1	0	9	4

23	2.5	0.25	Mod	7	0	49	8

24	2.5	0.5	Mod	6	0	2	12

25	5	0.25	Mod	40	0	89	8

26	5	0.5	Mod	20	0	46	7

27	7.5	0.25	Mod	79	0	96	4

28	7.5	0.5	Mod	47	0	65	5

29	10	0.25	Mod	81	0	99	5

30	10	0.5	Mod	60	0	78	9

Summary characteristics for the models simulated are listed, including the minor allele frequency simulated, the heritability of the simulated model, and the genetic model used. Complete penetrance functions are available from the authors upon request.

For the purely epistatic model, the XOR model, because of the range of power results seen, additional datasets were simulated with increased sample sizes. For each of the effect sizes, a total of 500 individuals (250 cases and 250 controls) were simulated. This resulted in an additional 10 models simulated, again with 100 replicate datasets simulated for each of these 10 models.

While power comparisons on a genome-wide scale are computationally infeasible in the scope of the current study, to evaluate the scalability of the GEDT method single datasets of different sizes were generated for timing experiments with GEDT. Using the XOR model described above, datasets with 500 cases and 500 controls were generated with a total of 1000, 10000, 100000, and 500000 total SNPs.

The simulated datasets generated in the current study are available by request directly from the authors or through http://www4.stat.ncsu.edu/~motsinger.

### Implementation

GEDT was used to analyze each of the simulated datasets described above. The configuration parameters used for analysis were as follows: 1000 generations, population size of 500 individuals, migration after every 25 generations, cross-over rate of 0.9, mutation rate of 0.01, chromosome size bounded by lower limit of 50 and upper limit of 1000, tournament type of selection, standard i.e. single-point cross-over, balanced accuracy for fitness evaluation, and sensible initialization. These parameters are all defined in the configuration file. To prevent stalling in local minima in the fitness landscape, the island model of parallelization is used where the population was split across 4 equally sized island populations. In this model, the best individual is passed to each of the other processes periodically (in this case, every 25 generations [36]). GEDT is implemented in C++ and was developed/tested on the Linux platform and data was analyzed on a quad-core Core2 Xeon processors (8 processors, each at 3 GHz and with 4GB of memory). Software and user instructions are available from the authors upon request, or linked from the following website: http://www4.stat.ncsu.edu/~motsinger.

The random search algorithm was used to analyze each of the 3000 simulated datasets as a negative control, and is implemented as a configuration parameter in the GEDT software. All configuration parameters used in the GEDT analysis were mirrored on the random search algorithm, with the exception of the parameters that control selection, mutation, and crossover. Population sizes and the number of generations were identical to those implemented in GEDT. The overall best random decision tree was selected as the final model.

For the purely epistatic XOR model simulations, C4.5 decision tree modelling was performed, as implemented in the J48 algorithm in freely available Weka software [37]. Default parameter settings were used, as follows: binarysplits = false, cinfidencefactor = 0.25, debug = false, minnumobj = 2, minfolds = 3, reducederrorpruning = false, saveinstancedata = false, seed = 1, subtreeraising = true, unpruned = false, uselaplace = false, and ten-fold cross-validation was used.

Power for all analyses was calculated in two ways. First, power was estimated under each epistasis model as the number of times the algorithm correctly identified both functional loci out of each set of 100 datasets, without any false positive or false negative loci. This represents a very conservative definition of power, where only models with only the exact simulated loci included in the final model contribute to the power estimates. Because of the conservative nature of this calculation, a more "liberal" definition of power was also considered. Under this definition, models that included only true positive loci (with or without false positive loci). Under this definition, models that correctly identified correct loci are considered a "success". While these definitions of power may not be traditional statistical definitions, they are intended as practical assessments of the performance of the methods.

### Power Results

The power results for each model are as shown in the Table 2. There are a few general trends that are expected for all association methods. First, as the effect size increases, the power of GEDT increases. Additionally, as the minor allele frequency increases, power generally increases. Also, the "liberal" power is always higher, as is expected since the results that count towards the "conservative" power estimates represent a subset of the models that contribute to the "liberal" estimate. Additionally, as is generally the case, the power to detect models with main effects is considerably higher than the purely epistatic model. Finally, as expected, the power of the random search is substantially lower than that of GEDT under both definitions, and is close to zero in most cases.

Because of the low power of GEDT to detect the XOR model, power was re-evaluated for larger sample sizes. The results are shown in Table 3. These results indicate that for increased sample sizes, GEDT has high power to detect purely epistatic interactions. Additionally, the power of C4.5 to detect purely epistatic models is included in Table 3. As expected, C4.5 is unable to model purely epistatic models due to the hierarchical model building approach used.

**Table 3:** Power Results for Purely Epistatic Simulated Models, n = 500

Model Number	Heritability (%)	Minor Allele Frequency	Genetic Model	Power(%)			Power (Lib %)		
				
				GEDT	Random Search	C4.5	GEDT	Random Search	C4.5
31	1	0.25	XOR	37	1	0	67	4	0

32	1	0.5	XOR	45	2	0	79	3	0

33	2.5	0.25	XOR	68	1	0	82	4	0

34	2.5	0.5	XOR	75	0	0	93	6	0

35	5	0.25	XOR	83	0	0	98	7	0

36	5	0.5	XOR	90	1	0	99	8	0

37	7.5	0.25	XOR	95	0	0	93	2	0

38	7.5	0.5	XOR	95	4	0	99	7	0

39	10	0.25	XOR	96	3	0	100	11	0

40	10	0.5	XOR	95	0	0	100	10	0

Summary characteristics for the models simulated are listed, including the minor allele frequency simulated, the heritability of the simulated model, and the genetic model used. Complete penetrance functions are available from the authors upon request.

The GEDT method is also computationally efficient, making it a reasonable approach for larger scale data analysis. For the simulated data used in the power calculations, the experiment described above was executed in parallel on a set of Linux blades with 2.33 Ghz processors, and each cross-validation replicate completed, on average, in under five minutes. To better understand the scalability of GEDT to large scale datasets, the results of the timing experiments for the larger SNP datasets are shown in Table 4. These results demonstrate that while the computation time is certainly not trivial, it is reasonable and feasible for genome-wide association study analysis.

**Table 4:** Analysis Times for Larger Datasets

Number of SNPs	Time for Analysis
1000	0.15

10000	0.5

100000	6.9

500000	33.9

Times are given in hours, for a single cross-validation interval

## Discussion

In the current study, we have presented a detailed description of a new methodology to detect gene-gene interactions in genetic association studies. We propose the use of grammatical evolution to evolve every aspect of decision tree models to detect gene-gene and gene-environment interactions. We demonstrate the potential of the method on a range of simulated two-locus gene-gene interaction models. GEDT has reasonably high power to detect models of small to moderate effect sizes, even in very small sample sizes as those simulated in the current study.

While these results are encouraging, the GEDT methodology is still in its infancy, and there are many aspects of its implementation and application that are currently under investigation. First, the parameters implemented in the configuration file are currently undergoing sweeps for a wide range of values to determine optimal settings for data analysis. For example, preliminary data (not shown) shows that as expected, as the number of generations that GEDT is run is increased, the power to detect models also increases. This trend should be further evaluated and potentially more sophisticated stopping rules should be considered. Other types of selection, different crossover and mutation rates, etc. should also be evaluated to maximize the power of the method.

Additionally, the performance of GEDT should be compared to other methods used in genetic epidemiology designed to detect epistasis - such as Multifactor Dimensionality Reduction [9], Grammatical Evolution Neural Networks [13], etc. Also, the performance of GEDT should be further compared to other decision tree algorithms and alternate implementations of the C4.5 algorithm implemented in the current study [22]. No method can be considered in a vacuum - and empirical comparisons will play an important role in understanding the niche of the GEDT methodology. These comparisons should consider a variety of genetic models, including heterogeneity, the presence of phenocopy, etc.

Additionally, alternate approaches to internal model validation should be explored for the GEDT algorithm that might allow more than a single final best model to be evaluated. Other highly successful decision tree approaches, such as Random Forest [11] use bootstrapping approaches to rank attributes in order of importance. Considering extensions of GEDT inspired by such approaches will be an important future research direction.

The current results indicate the potential of this exciting new approach, but as the end goal of any methodological development is the application to real data, GEDT should be applied to real datasets in human genetics to really evaluate its potential.

## Conclusions

In the current study, we introduce a new approach for detecting gene-gene interactions in genetic association studies, a grammatical evolution optimized decision tree approach (GEDT). GEDT is able to detect interactions in the presence and the absence of main effects. We demonstrate the potential of the method in a range of simulated data; GEDT has high power to detect genetic risk models of very low effect sizes in relatively small samples. GEDT is a promising new approach for human genetics.

## Competing interests

The authors declare that they have no competing interests.

## Authors' contributions

AMR conceived of the current study and contributed to writing the manuscript. SD worked on the coding and implementation of the GEDT method and contributed to writing the manuscript. SJW simulated the data used for the experiment, and contributed to writing the manuscript. NEH contributed to the coding and implementation of GEDT method and performed the data analysis. All authors have read and approved the final manuscript.

## References

[B1] Altshuler D Daly MJ Lander ES Genetic mapping in human disease Science 2008 322 881 888 10.1126/science.1156409 PMC2694957 18988837

[B2] Moore JH Ritchie MD STUDENTJAMA. The challenges of whole-genome approaches to common diseases JAMA 2004 291 1642 1643 10.1001/jama.291.13.1642 15069055

[B3] Hirschhorn JN Genomewide association studies--illuminating biologic pathways N Engl J Med 2009 360 1699 1701 10.1056/NEJMp0808934 19369661

[B4] Goldstein DB Common genetic variation and human traits N Engl J Med 2009 360 1696 1698 10.1056/NEJMp0806284 19369660

[B5] Moore JH The ubiquitous nature of epistasis in determining susceptibility to common human diseases Hum Hered 2003 56 73 82 10.1159/000073735 14614241

[B6] Bellman R Adaptive Control Processes 1961 Princeton: Princeton University Press

[B7] Moore JH Williams SM New strategies for identifying gene-gene interactions in hypertension Ann Med 2002 34 88 95 10.1080/07853890252953473 12108579

[B8] Motsinger AA Ritchie MD Reif DM Novel methods for detecting epistasis in pharmacogenomics studies Pharmacogenomics 2007 8 1229 1241 10.2217/14622416.8.9.1229 17924838

[B9] Ritchie MD Hahn LW Roodi N Bailey LR Dupont WD Parl FF Moore JH Multifactor-dimensionality reduction reveals high-order interactions among estrogen-metabolism genes in sporadic breast cancer Am JHum Genet 2001 69 138 147 10.1086/321276 PMC1226028 11404819

[B10] Nelson MR Kardia SL Ferrell RE Sing CF A combinatorial partitioning method to identify multilocus genotypic partitions that predict quantitative trait variation Genome Res 2001 11 458 470 10.1101/gr.172901 PMC311041 11230170

[B11] Brieman L Random Forests Machine Learning 2001 45 27

[B12] Aguilar-Ruiz JS Moore JH Ritchie MD Filling the gap between biology and computer science BioData Min 2008 1 1 10.1186/1756-0381-1-1 PMC2547862 18822148

[B13] Motsinger-Reif AA Dudek SM Hahn LW Ritchie MD Comparison of approaches for machine-learning optimization of neural networks for detecting gene-gene interactions in genetic epidemiology Genet Epidemiol 2008 10.1002/gepi.20307 18265411

[B14] Yao X Evolutionary artificial neural networks Int J Neural Syst 1993 4 203 222 10.1142/s0129065793000171 8293227

[B15] Motsinger-Reif AA Ritchie MD Neural networks for genetic epidemiology: past, present, and future BioData Min 2008 1 3 10.1186/1756-0381-1-3 PMC2553772 18822147

[B16] Koza J Rice JP Genetic generation of both the weights and architecture for a neural network IEEE Transactions 1991 2

[B17] Moore JH Parker JS Olsen NJ Aune TM Symbolic discriminant analysis of microarray data in autoimmune disease Genet Epidemiol 2002 23 57 69 10.1002/gepi.1117 12112248

[B18] Moore JH Andrews PC Barney N White BC Development and evaluation of an open-ended computational evolution system for the genetic analysis of susceptibility to common human diseases Lecture Notes in Computer Science 2008 4973 11

[B19] Greene CS White BC Moore JH Ant colony optimization for genome-wide genetic analysis Lecture Notes in Computer Science 2008 5217 10

[B20] O'Neill M Ryan C Grammatical Evolution 2001 Boston: Kluwer Academic Publishers

[B21] O'Neill M Ryan C Grammatical Evolution: Evolutionary automatic programming in an arbitrary language 2003 Boston: Kluwer Academic Publishers

[B22] Alpaydin E Introduction to Machine Learning 2004 Cambridge, MA: MIT Press

[B23] Shepherd BA An appraisal of a decision-tree approach to image classification Proceedings of the Eighth International Joint Conference on Artificial Intelligence 1983 2

[B24] Devroy LGL Lugosi G A Probabilistic Theory of Pattern Recognition 1996 New York: Springer

[B25] Velez DR White BC Motsinger AA Bush WS Ritchie MD Williams SM Moore JH A balanced accuracy function for epistasis modeling in imbalanced datasets using multifactor dimensionality reduction Genet Epidemiol 2007 31 306 315 10.1002/gepi.20211 17323372

[B26] Hastie TJ Tibshirani RJ Friedman JH The elements of statistical learning 2001 Basel: Springer Verlag

[B27] Koza J Genetic Programming: on the programming of computers by means of natural selection 1992 Cambridge, MA: MIT Press

[B28] Miller BLG D E Genetic Algorithms, Tournament Selection and the Effects of Noise Complex Systems 1995 9 193 212

[B29] Quinlan JR Programs for Machine Learning 1993 Morgan Kaufmann Publishers

[B30] Witten IH Frank E Programs for Machine Learning 2005 2 Morgan Kaufmann

[B31] Cordell HJ Epistasis: what it means, what it doesn't mean, and statistical methods to detect it in humans Hum Mol Genet 2002 11 2463 2468 10.1093/hmg/11.20.2463 12351582

[B32] Li W Reich J A complete enumeration and classification of two-locus disease models Hum Hered 2000 50 334 349 10.1159/000022939 10899752

[B33] Neuman RJ Rice JP Two-locus models of disease Genet Epidemiol 1992 9 347 365 10.1002/gepi.1370090506 1427023

[B34] Culverhouse R Suarez BK Lin J Reich T A perspective on epistasis: limits of models displaying no main effect Am J Hum Genet 2002 70 461 471 10.1086/338759 PMC384920 11791213

[B35] Dudek SM Motsinger AA Velez DR Williams SM Ritchie MD Data simulation software for whole-genome association and other studies in human genetics Pac Symp Biocomput 2006 499 510 17094264

[B36] Cantu-Paz E Evolving Neural Networks for the classification of galaxies 2002 San Franscisco: Morgan Kaufman Publishers

[B37] Hall M Frank E Holmes G Pfahringer B Reutemann P Witten IH The WEKA Data Mining Software: An Update SIGKDD Explorations 2009 11

